# Comparison Between the Mental Health of Mothers of Children With Autism and Control Group

**Published:** 2012

**Authors:** Forough Riahi, Sakineh Izadi–mazidi

**Affiliations:** 1 Ahvaz Jundishapur University of Medical Sciences, Ahvaz, Iran.; 2 Shahid Chamran University, Ahvaz, Iran

**Keywords:** Autism, Children, Mental health, Mother

## Abstract

**Objective:** The aim of this study was to compare the mental health of mothers of children with autism and those of control group.

**Methods**: Sample of this study consists of 32 mothers of Children with Autistic disorder and 29 mothers of children without Autism; referring to hospitals of Ahvaz city using Convenience sampling. All subjects were asked to complete Demographic questionnaire and General HealthQuestionnaire (GHQ). Data were analyzed using Multivariate Analysis of variance (MANOVA).

**Results**: Resultsindicated that significant differences exist in mental health (F=5.69, P=0.02) and anxiety/ insomnia (F=4.82, P=0.03), between mothers of children with Autismand control group. There were not any other significant differences in the other subscales.

**Conclusion:** It is essential using some mental health improving interventions for mothers of children with Autistic disorder.

## Introduction

Parents’ mental health could beintensivelyinfluenced bydisabled child, especially when there isa disordersuch as Autism including a wide range of behaviors and particularly social behaviors (1). Autistic disorder is one of the pervasive developmental disorders characterized by delay and deviance in the development of social, communicative and other skills, various motor mannerisms, resistance to change, and idiosyncratic interests and preoccupations(2). Existing studies suggest that characteristics of the disorder cause stress in parents. And the more severe the child’s symptoms, the greater will be the degree of parental stress (3). And some studies found that They often experience frustration and pessimism, and they have particular characteristics such as schizoid traits, higher traits of aloof, hypersensitive, anxious, tense, and rigid which in turn contribute to their increased psychological stress (4,5). It also has been reported that parents of children with autism experience more marital distress and conflicts with non-disabled children (6).Sharply et al state that mothers experience greater impact than fathers (4). They blame themselves for their children’s disorder, more likely than mothers of normativechildren (6). They are also the parent who was most likely to be held responsible for their child’s behavior (7).

Havingan autistic child has a remarkable effect on mother's careers. Those who are able to work are often forced to miss work, performing below their normal level orgetting back to part-time status. Resentment is observed among mothers caused by this problem (3).

So it is necessary to study mother’s problems and their psychological needs (because mothers are the family members with the most connection with these children).More knowledge about the adaptation process and development of psychopathologies in parents will let us design more effective strategies and programs to help them (5). But few studies have been performed in Iran (1).The purpose of this study was to compare the mental health of mothers of children with Autism to mothers of children without autism.

## Materials and Methods

Sample of this Study included 32 mothers with autistic children who referred to child and adolescent psychiatrist, and 29 mothers without children autism referred to pediatrician in Ahvaz city hospitals. Two groups were selectedby convenience sampling and were matched based on marital status, occupational status, level of education and ethnicity (Table1).Children with autistic disorder were diagnosed by a child and adolescent psychiatrist, according to DSMIV-TR criteria. And children without autism didn’t suffer from any physical or mental illness.

Data was gathered using GHQ. The questionnaire presented by Goldberg and Hillary for screening of non-psychotic psychological disorders, in 1979. It has 28 questions and four sub-scales and each scale has 7 questions. Adham et al evaluated validity of test 91% and 88% respectively. Reliability was calculated by Cronbach’s alpha as ameasure of internal consistency reliability. Cronbach’s alpha was 0.84 for physical symptoms, social functioning 79%, depression 81% and mental health 91% (8).

Noorbala, Bagheriyazdi, Mohammad (2009) evaluated the concurrent validity of Persian form of the questionnaire by the Symptom Checklist- 90- Revised. They found that there was a significant correlation between scores of samples within the subscales of depression(r=0.72), Anxiety(r=0.75), physical symptoms(r=0.67) and total score(r=0.84) of these instruments (p<0.001). The evaluation of reliability of GHQ-28 carried out by the test- retest procedure a week after the first stage. The correlation was significant(r=0.85, p<0.01) (9). Demographic data questionnaire was also filled in.

Data were analyzed using MANOVA.The probability level of 0.05 was accepted statistically significant. Statistical analyses were carried out by SPSS software, version16.

## Results

Mean (±SD) age of mothers with and withoutautistic childrenwere 34.12(±5.9) and 37.27(±5.2) respectively (p=0.09). Frequencies of the mothers’ demographic features are listed in table 1. 

**Table1:** Frequencies of the mothers’ demographic features

Feature	Frequency		P-value
	mothers with autistic children	mothers without autistic children	
**Marital status** Married Divorced/widowed	32(100%) 0	29(100%) 0	-
**Occupation status** Yes No	2(7%) 30(93%)	1(3%) 28(97%)	0.62
**Level of education** High school Diploma Bachelor Post graduate	6(19%) 19(59%) 6(19%) 1(3%)	7(24%) 17(59%) 4(14%) 1(3%)	0.33
**Ethnicity** Fars Arab Lor Other	16(50%) 7(22%) 9(28%) 0	18(62%) 5(17%) 6(21%) 0	0.43

Tables 2, shows the mean and Standard deviation of mental health variable and its subscales in mothers with and without autistic children; and results of MANOVA.

As shown in table 2, there are significant differences between mothers with and without autistic children in mental health variable (F=5.69, P=0.02) and its subscale, anxiety/ insomnia (F=4.82, P=0.03). There are not any other significant differences in the other subscales.

**Table2:** Mean and SD of mental health variable and its subscales in mothers with and without autistic children; and results of MANOVA

**Variable**	**Mean(± SD)**		**df**	**Mean Square**	**F**	**P-value**
	**mothers with autistic children**	**mothers without autistic children**				
Mental health	58.71± 13.29	51.34± 10.51	1	827.20	5.69	0.02
physical symptoms	13.96± 4.40	13.20± 3.74	1	8.83	0.52	0.47
Anxiety/insomnia	16.78± 8.21	13.03± 4.30	1	213.56	4.82	0.03
social dysfunction	17.62± 2.56	16.78± 3.05	1	10.72	1.33	0.25
Depression	15.65± 5.31	14.68± 5.05	1	14.21	0.52	0.47

## Discussion

Results indicated that mothers of children diagnosed with Autism had significantly lower mental health and experiencemore anxiety /insomnia than mothers of children without autism. 

This finding was consistent with some previous research, such as Tarabek, Nouri, Yamada et al, and Salehi et al.’s study who found that mothers of children with Autism and other disabilities reported significantly less mental health than mothers of children with control group (6, 1, 10, 11).

McCarthy et al and Gray also found that mothers of children with disability experiencea lotof stress (12, 13). Firat et al reported a significant increase on anxiety- tense in mothers of autism group in comparison to those of mental retarded children(5). These are similar to our finding that the mothers experience anxiety/ insomnia more than control group. 

In explanation of the results it could be stated that frustration of having a disabled child, Maladaptive behaviors that characterize autism, guilt feeling resulting from anger and rejection of the child, being not aware of the causes of autism, and unknown cure, contending with the stigmatization associated with the disorder, additional expenses which can create financial burden, restrictions on normal lifestyle, lifetime care, worrying about the future of children and blaming mother as being responsible for disorder, threat mental health of family members and cause anxiety, especially in mothers(3,14-16).

However, there aren’t significant differences in other subscales- Social dysfunction, Depression, Physical symptoms. These finding areconsistent with results of some studies (16, 17)which have not found differences in depression between mothers ofchildren with special needs compared to norms or to control groups.Our results are in contrast with some other studies(1, 5, 18,19).

Perhaps this difference from the existing literature was due in part to the relatively small sample size of mothers of children with Autism in this study, or it is possible that the mothers of children with autism in the study were receiving more social support, which could decrease their complaints about social dysfunction, depression and physical symptoms. This may be due to our Iranian cultural system and support from family. 

## Conclusion

 The diagnosis of autism affects the parent’s life. The nature of the disability might affect psychological well-being of the parents. So clinicians should be aware of this issue. And don’t focus on the child, only.

## Limitations

 Our research has some limitations; thisstudyexamineda small sample, so the findings must be generalized with caution. Also only mothers took part in the study. 

It is suggested to on larger groups of samples that will also focus on the fathers’ and siblings’ distress as well as those of mothers. It is also recommended to provide someprograms to support families (especially mothers) that can strengthen family coping and positive adjustment. 

## Authors’ contributions

FR and SI-M conceived and designed the evaluation, collected the clinical data, performed the statistical analysis and interpreted them. SI-M drafted the manuscript and FR revised it. Both authors read and approved the final manuscript. 

## Notes

**Declaration of interest:** None.
